# Interference Mitigation Using UNet for Integrated Sensing and Communicating Vehicle Networks via Delay–Doppler Sounding Reference Signal Approach

**DOI:** 10.3390/s25061902

**Published:** 2025-03-19

**Authors:** Yuanqi Tang, Yu Zhu

**Affiliations:** School of Information Science and Technology, Fudan University, Shanghai 200438, China; 22210720071@m.fudan.edu.cn

**Keywords:** ISAC, vehicle network, sounding reference signals, deep learning

## Abstract

Advanced communication systems, particularly in the context of autonomous driving and integrated sensing and communication (ISAC), require high precision and refresh rates for environmental perception, alongside reliable data transmission. This paper presents a novel approach to enhance the ISAC performance in existing 4G and 5G systems by utilizing a two-dimensional offset in the Delay–Doppler (DD) domain, effectively leveraging the sounding reference signal (SRS) resources. This method aims to improve spectrum efficiency and sensing accuracy in vehicular networks. However, a key challenge arises from interference between multiple users after the wireless propagation of signals. To address this, we propose a deep learning-based interference mitigation solution using an UNet architecture, which operates on the Range–Doppler maps. The UNet model, with its encoder–decoder structure, efficiently filters out unwanted signals, therefore enhancing the system performance. Simulation results show that the proposed method significantly improves the accuracy of environmental sensing and resource utilization while mitigating interference, even in dense network scenarios. Our findings suggest that this DD-domain-based approach offers a promising solution to optimizing ISAC capabilities in current and future communication systems.

## 1. Introduction

Next-generation mobile communication systems, including 5G-A and 6G, must support a wide range of demanding services, such as autonomous driving and real-time virtual reality [1]. These applications require ultra-low latency, high data throughput, and the ability to connect numerous devices with high reliability and security. In autonomous driving, radar sensing plays a vital role by providing critical environmental awareness for vehicles. At the same time, Vehicle-to-Everything (V2X) networks are expected to deliver low-latency and high-capacity data communications, even in highly dynamic environments [2]. As the frequency bands for communication and radar sensing increasingly overlap, the integration of both functions within the same spectrum, i.e., integrated sensing and communication (ISAC), has become a focal point for advancing next-generation V2X technologies [3,4,5].

A major challenge in achieving ISAC in V2X networks is ensuring that multiple users can coexist while simultaneously receiving both high-precision sensing and efficient communication services. The need for effective ISAC is especially urgent as the number of smart vehicles and connected devices grows, further straining the limited spectrum resources. Although recent studies have explored active sensing within ISAC systems [6,7,8,9], they typically rely on pilot-based methods within limited sub-bands for communications, which are often inadequate for vehicular radar sensing. While the sounding reference signal (SRS) has been identified as a potential resource for enabling broader sensing capabilities, the concurrent multi-user sensing in the SRS channel has not been fully explored. This is particularly challenging when inter-user interference (IUI) arises due to the shared time–frequency (TF) resources in the system.

The emergence of Orthogonal Time Frequency Space (OTFS) technology has sparked interest in two-dimensional (2D) pilot designs. For instance, reference [10] reduces the guard interval between pilot and data symbols, optimizing pilot sequences and power allocation to maximize spectral efficiency while ensuring accurate channel estimation. Similarly, reference [11] addresses pilot placement in the Delay–Doppler (DD) domain to minimize the mean square error (MSE) of channel interpolation and balance power allocation between pilot and data symbols. Considering the application in ISAC systems, reference [12] proposes an information-theory-based target detection framework that utilizes a relative entropy test and an iterative waveform design method with OTFS signals, which improves the detection performance. Despite their contributions, these studies primarily focus on optimizing pilot placement and power distribution in the DD domain, neglecting the challenges posed by integrating multiple sounding signals and mitigating the IUI in ISAC systems with limited resources. Additionally, the implementation of OTFS technology requires significant modifications to existing systems with a new waveform introduced.

Interference cancellation using neural networks is another significant area of research that leverages the 2D characteristics of TF channels. For example, reference [13] exploits properties of the angle-delay and spatial frequency domains using a dual CNN architecture, improving multi-user channel estimation under time-varying conditions and pilot contamination across base stations (BSs). Reference [14] combines model-based and non-model-based deep neural networks (DNNs) in a cascaded structure, employing online Bayesian learning to adaptively mitigate interference in real-time scenarios. Additionally, OTFS technology has inspired image-processing-based methods, such as CNNs for feature extraction in OTFS multiple-access systems [15] and DNNs for reducing computational complexity in channel estimation in the DD domain [16]. However, these methods often neglect the complexities of dynamic vehicular networks and the challenges posed by non-orthogonality between multiple users in ISAC environments.

Inspired by [17], which emphasizes leveraging the limited local environment and TF modulation of SRS sequences in ISAC systems, we propose a novel DD-SRS-based (DD-domain-SRS-based) multi-user ISAC framework. This approach incorporates a 2D image-neural-network-based scheme to extract local environment information, conserving valuable TF resources while accommodating more users compared to orthogonal resource allocation frameworks [17,18,19] within constrained TF scopes to address the challenges of increasingly dense network environments. It is noted that a scheme based on Range–Doppler maps (RD maps) and image segmentation is investigated in [20,21] for multi-target signal classification. To the best of our knowledge, the application of IUI mitigation in non-orthogonal multiple-access scenarios has received limited attention.

Our primary contributions are as follows:

- Proposing a novel solution for ISAC technology in V2X networks: We introduce an innovative approach that leverages the 2D offset in the DD domain within existing 4G/5G systems. This method maximizes the utilization of SRS for both radar sensing and communications, addressing the increasing demand for high-precision and high-refresh-rate environmental perception in autonomous driving and other V2X applications.
- Addressing multi-user interference in ISAC systems: We recognize the challenge of the IUI when multiple users share limited TF resources in the SRS channel. To mitigate the IUI, we propose a deep learning (DL)-based scheme using a UNet architecture, which effectively reduces interference and enhances the accuracy of sensing in multi-user scenarios.
- Demonstrating the feasibility and effectiveness of the proposed method: Through extensive simulations, we validate the proposed method’s capability to deliver robust and reliable multi-user sensing and communications in realistic V2X environments. The results show significant improvements in the system performance, with reduced interference and enhanced sensing accuracy, making the proposed solution viable for next-generation autonomous vehicle networks.

The remainder of this paper is organized as follows: Section 2 introduces the considered system model and ISAC signal models. Section 3 describes the proposed DD-SRS-based framework and its design principles, as well as the 2D neural network scheme, emphasizing the role of pixel segmentation in extracting local environment information. Section 4 presents experimental results and performance evaluations, demonstrating the effectiveness of the proposed approach in multi-user ISAC scenarios. Finally, Section 5 concludes the paper with a summary of key findings and suggestions for future research.

Notations: $j=\sqrt{-1}$ denotes the imaginary unit. A, a and *a* represent a matrix, a column vector and a scalar, respectively. Conjugate transpose and transpose operators are denoted by ${\left(\cdot \right)}^{H}$ and ${\left(\cdot \right)}^{T}$, respectively. $C^{M\times N}$ represents the set of all M×N complex-valued matrices. CN(0,K) denotes the circularly symmetric complex Gaussian distribution with zero mean and covariance matrix K.

## 2. System and Signal

**Figure 1:**
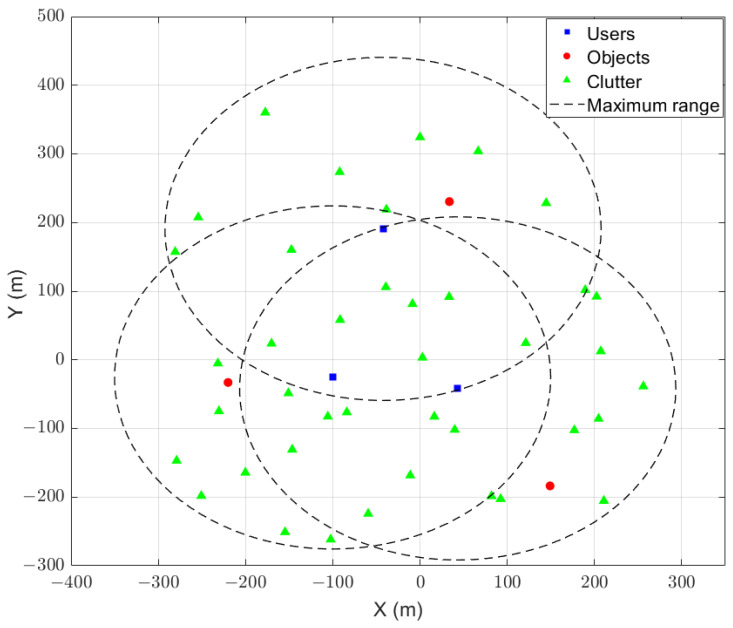
ISAC V2X scenario for 3 users.

In this paper, we investigate ISAC-assisted multi-user V2X networks with orthogonal frequency division multiplexing (OFDM) technology. A BS simultaneously serves *K* users indexed by k∈K≜{0,...,K−1} within a cell, utilizing *N* OFDM subcarriers and *M* consecutive symbols for sensing purposes. To maintain compatibility with existing 4G/5G V2X communication systems, it is assumed that each vehicle is equipped with ISAC devices employing OFDM signals, enabling both sensing and communication functionalities concurrently. To ensure precise sensing in high-mobility environments, each user fully exploits the TF resources of the uplink SRS sent to the BS for sensing operations. Full-duplex transmission and reception are facilitated by physically separated transmitting and receiving antennas equipped by vehicles. While transmitting the uplink SRS to the BS to acquire channel state information (CSI), each user also leverages the reflected signals to sense the surrounding environment. However, the echoes are interfered by signals from other users within the cell, which can obscure targets of interest and thereby lead to missed detections, or cause an increase in false alarm probability due to the superposition of signals. As illustrated in Figure 1, our simulated ISAC V2X network environment features three randomly moving users, each with a distinct target of interest amidst a stationary clutter background. Thus, this setup highlights the dual role of communication and sensing in overcoming the challenge of IUI and enhancing the accuracy of environmental perception.

The baseband signal for the *k*-th user in the time domain is represented by

(1) $$s_{k}\left(t\right)=\sum_{m=0}^{M-1}s_{k,m}\left(t\right),$$

where $s_{k,m}\left(t\right)$ represents the OFDM signal in the *m*-th symbol for the *k*-th user and is given by

(2) $$s_{k,m}\left(t\right)=\frac{1}{\sqrt{N}}\sum_{n=0}^{N-1}x_{k,n,m}e^{j2\pi n\Delta ft}\mathrm{rect}\left(\frac{t-mT_{sym}}{T_{sym}}\right),$$

where $x_{k,n,m}$ represents the symbol of the transmit SRS at the *n*-th subcarrier in the *m*-th symbol for the *k*-th user in the frequency domain with M,N denoting the number of symbols and subcarriers of SRS signals transmitted. Δf denotes the subcarrier spacing and $T_{sym}≜T_{s}+T_{CP}$ is the total OFDM symbol duration time, where $T_{s},T_{CP}$ denotes the effective OFDM symbol duration and the cyclic prefix (CP) duration, respectively.

After encountering the scattering from the surrounding environments, and considering the interference from other users, the baseband signal received by the *k*-th user in the time domain [17,22] is represented by

(3) $$\begin{array}{cc} y_{k}\left(t\right) & =\sum_{l=0}^{L_{k}-1}\alpha_{k,l}s_{k}\left(t-\tau_{k,l}\right)e^{-j2\pi f_{c}\tau_{k,l}}e^{j2\pi \nu_{k,l}t}\\ \; & \;+\sum_{i=0,i\neq k}^{K-1}\sum_{l=0}^{L_{i}-1}\alpha_{i,l}s_{i}\left(t-\tau_{i,l}\right)e^{-j2\pi f_{c}\tau_{i,l}}e^{j2\pi \nu_{i,l}t}+w_{k}\left(t\right), \end{array}$$

where $L_{k}$ is the total number of targets and clutter in the local environment of the *k*-th user, and $\alpha_{k,l},\tau_{k,l},\nu_{k,l}$ is the attenuation coefficient, the time delay and the Doppler frequency offset of the *l*-th path for the *k*-th user, respectively. $w_{k}\left(t\right)∼\mathrm{CN}\left(0,\sigma_{k}^{2}\right)$ denotes the additive white Gaussian noise with variance $\sigma_{k}^{2}$. Furthermore, by denoting $\tau_{k,\max},\nu_{k,\max}$ as the maximum round-trip delay and the maximum Doppler shift of targets and clutter in the local environment of the *k*-th user, respectively, it is assumed that $T_{CP}\geq \tau_{k,\max}$ [22,23] and that $\nu_{k,\max}T_{sym}\ll 1$ [22,24].

After the CP removal, the inverse Fast Fourier Transforming (IFFT) operation, and time-domain discrete sampling, the received signal within *M* symbols $Y_{k}\in C^{N\times M}$ of the *k*-th user [22] is given by

(4) $$\begin{array}{cc} Y_{k} & =\sum_{l=0}^{L_{k}-1}\alpha_{k,l}F_{N}^{H}\left(X_{k}⊙\left(b\left(\tau_{k,l}\right)c^{H}\left(\nu_{k,l}\right)\right)\right)\\ \; & \;+\sum_{i=0,i\neq k}^{K}\sum_{l=0}^{L_{i}-1}\alpha_{i,l}F_{N}^{H}\left(X_{i}⊙\left(b\left(\tau_{i,l}\right)c^{H}\left(\nu_{i,l}\right)\right)\right)+W_{k}, \end{array}$$

where $X_{k}\left[n,m\right]=x_{k,n,m}$ and $F_{N}^{H}\in C^{N\times N}$ denotes the inverse Discrete Fourier Transforming (IDFT) matrix. $b\left(\tau \right),c\left(\nu \right)$ are given by

(5) $$\begin{array}{cc} \; & b\left(\tau \right)={\left[\begin{array}{c} 1,e^{-j2\pi \Delta f\tau },\ldots ,e^{-j2\pi (N-1)\Delta f\tau } \end{array}\right]}^{T},\\ \; & c\left(\nu \right)={\left[\begin{array}{c} 1,e^{-j2\pi \nu T_{\mathrm{sym}}},\ldots ,e^{-j2\pi \left(M-1\right)\nu T_{\mathrm{sym}}} \end{array}\right]}^{T}. \end{array}$$

In this paper, we focus on the local sensing environment information (local SEI) in the frequency domain as the primary information of interest for each user within the ISAC V2X system. The local SEI of the *k*-th user is given by

(6) $$H_{k}=\sum_{l=0}^{L_{k}-1}\alpha_{k,l}\left(b\left(\tau_{k,l}\right)c^{H}\left(\nu_{k,l}\right)\right).$$

## 3. Framework and Scheme

**Figure 2:**
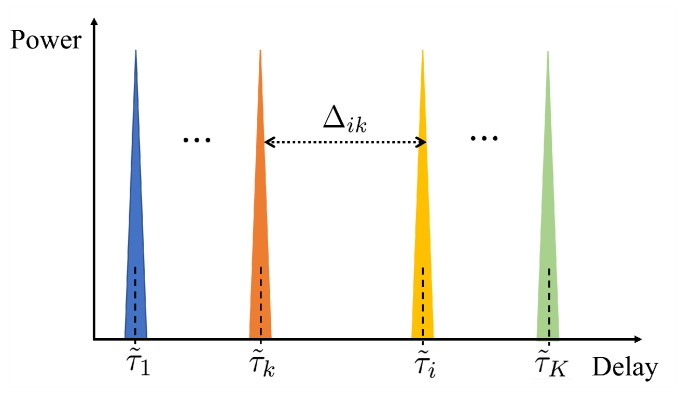
Orthogonal SRS in delay domain.

In this section, we introduce the DD-SRS-based ISAC-assisted multi-user framework and the DL-based interference cancellation scheme based on the system discussed in Section 2. In long-term evolution (LTE) systems, the BSs utilize perfect orthogonal sequences, such as Zadoff–Chu (ZC) sequences, as multi-user uplink SRS sequences based on their orthogonality in the delay domain [25,26]. As shown in Figure 2, where each colored peak represents an SRS sequence for a distinct user, multiple users sharing identical TF resource blocks are separated based on the orthogonality of SRS sequences in the delay domain. In Figure 2, ${\tilde{\tau }}_{k},k\in K$ is the introduced phase offset of the SRS sequence in the delay domain for the *k*-th user and $\Delta_{ik}≜{\tilde{\tau }}_{i}-{\tilde{\tau }}_{k}$ denotes the interval of phase shifts between the *i*-th user and the *k*-th user in the delay domain. It is noted that the delay depicted in Figure 2 does not represent the actual channel propagation delay, but rather the designed offset of the SRS sequence in the time or frequency domain.

In our paper, we exploit the orthogonality of SRS in the DD domain to expand the capacity of multiple access of ISAC V2X systems. However, the orthogonality can be contaminated in the DD domain due to the high mobility of users, introducing IUI to the RD maps. To mitigate the IUI, we propose the image-processing-based and DL-based interference cancellation scheme. The steps of our overall scheme are as follows: Firstly, we design the SRS in the DD domain for multiple users, and construct their respective SEI. Secondly, based on the randomly generated SRS and the received signal model in (4), we generate the original RD maps for each user that involve their local SEI and the IUI terms from other users. Thirdly, we train a deep neural network based on image segmentation to extract each user’s local SEI in the DD domain from the original RD maps. Finally, we utilize digital filtering and the IFFT operation to reconstruct the SEI for each user based on the information extracted. It is worth noting that since the local SEI can also serve as the communication CSI, the proposed multiple-access framework and interference mitigation scheme are designed for both channel estimation and vehicle sensing, indicating that the communication and sensing functionalities are aligned.

### 3.1. Multiple-Access ISAC Framework Based on DD-SRS

We first observe the conditions that the local SEI can be perfectly estimated with the traditional SRS sequences, which are orthogonal in the delay domain in LTE systems. The *N*-point SRS sequence for the *k*-th user in the *m*-th symbol in the frequency domain [26] is given by

(7) $$x_{k,m}\left[n\right]=x_{b,m}\left[n\right]e^{j2\pi \left(n-1\right)\Delta f{\tilde{\tau }}_{k}},$$

where $x_{b,m}$ is an SRS sequence in the *m*-th symbol, which serves as a base sequence. Based on the received signal matrix in the TF domain, the estimated local SEI for the *k*-th user is

(8) $$\begin{array}{cc} {\hat{H}}_{k}\; & =\left(F_{N}Y_{k}\right)⊙X_{k}^{*}\\ \; & =\sum_{l=0}^{L_{k}-1}\alpha_{k,l}b\left(\tau_{k,l}\right)c^{H}\left(\nu_{k,l}\right)+\sum_{\begin{array}{c} i=0,i\neq k \end{array}}^{K}\sum_{l=0}^{L_{i}-1}\alpha_{i,l}b\left(\tau_{i,l}+\Delta_{ik}\right)c^{H}\left(\nu_{i,l}\right)+\left(F_{N}W_{k}\right)⊙X_{k}^{*}. \end{array}$$

**Figure 3:**
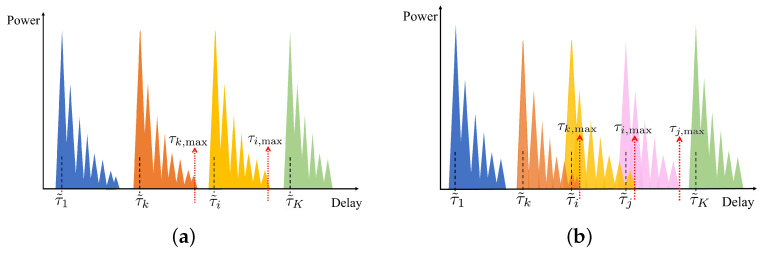
Multiple access based on one-dimensional SRS: (**a**) orthogonal multiple access; (**b**) non-orthogonal multiple access.

It can be seen that the desired local SEI of the *k*-th user $\sum_{l=0}^{L_{k}-1}\alpha_{k,l}b\left(\tau_{k,l}\right)c^{H}\left(\nu_{k,l}\right)$ can be estimated perfectly through digital filtering, i.e., extracting the parts within $0\leq \tau \leq \tau_{k,\max}$ in the delay domain, when $\Delta_{ik}>\tau_{k,\max},\forall i\in K$. That is, as long as the delay interval between any two users exceeds the maximum multi-path delay spread, the IUI component can be perfectly mitigated, allowing the BS to accurately estimate the CSI for multiple users, as illustrated in Figure 3a. However, when time-domain resources are more severely constrained due to more users, the requirement $\Delta_{ik}>\tau_{k,\max}$ to maintain the SRS orthogonality is harder to satisfy, leading to strong IUI, as shown in Figure 3b. Therefore, this method of maintaining SRS orthogonality in the one-dimensional delay domain limits the capacity for multiple access.

**Figure 4:**
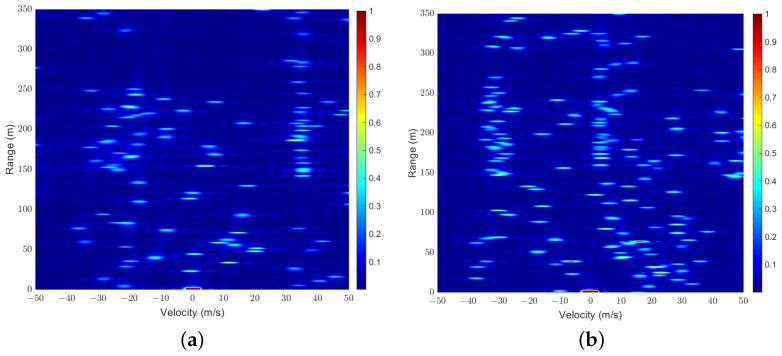
RD map based on the DD-SRS: (**a**) orthogonal multiple access; (**b**) non-orthogonal multiple access.

Inspired by [17], we leverage the properties of signals in the DD domain to construct multi-user SRS, thereby facilitating the efficient separation of signals of different users and expanding the multi-user capacity. This DD-SRS matrix in the frequency domain for the *k*-th user is given by

(9) $$X_{k}\left[n,m\right]=x_{b,m}\left[n\right]e^{j2\pi \left(n-1\right)\Delta f{\tilde{\tau }}_{k}}e^{-j2\pi \left(m-1\right){\tilde{\nu }}_{k}T_{\mathrm{sym}}},$$

where ${\tilde{\nu }}_{k}$ denotes the offset of the SRS in the Doppler domain for the *k*-th user. We adopt this SRS to satisfy the sensing needs of users sharing identical resource blocks. Similar to (8), the estimated local SEI with the DD-SRS in the frequency domain is given by

(10) $$\begin{array}{cc} {\hat{H}}_{k}\; & =H_{k}+{\tilde{H}}_{k}+{\tilde{W}}_{k}\\ \; & =\sum_{l=0}^{L_{k}-1}\alpha_{k,l}b\left(\tau_{k,l}\right)c^{H}\left(\nu_{k,l}\right)\\ \; & \;+\sum_{\begin{array}{c} i=0,i\neq k \end{array}}^{K}\sum_{l=0}^{L_{i}-1}\alpha_{i,l}b\left(\tau_{i,l}+\Delta_{ik}\right)c^{H}\left(\nu_{i,l}+\delta_{i,k}\right)+\left(F_{N}W_{k}\right)⊙X_{k}^{*}, \end{array}$$

where $\Delta_{ik}={\tilde{\tau }}_{k}-{\tilde{\tau }}_{i},\delta_{i,k}={\tilde{\nu }}_{k}-{\tilde{\nu }}_{i}$ represents the interval of phase of the utilized DD-SRS between the *i*-th user and the *k*-th user in the delay and the Doppler domain, respectively. For the simplicity of expression, $H_{k}$ denotes the desired local SEI as given in (8), and ${\tilde{H}}_{k},{\tilde{W}}_{k}$ denotes the IUI SEI and the noise term, respectively. Assuming the perfect time and frequency synchronization at the transceiver of user *k*, ${\tilde{H}}_{k}$ can only be eliminated when the intervals satisfy the requirement that

(11) $$\Delta_{ik}>\tau_{k,\max}\;\mathrm{or}\;\delta_{ik}>\nu_{k,\max}+\nu_{i,\max},\forall i\in K,i\neq k,$$

where $\tau_{k,\max},\nu_{k,\max}$ denotes the maximum delay and the Doppler shift of multiple paths of the user *k*. The mathematical proof of the condition of orthogonality in (11) is given in Appendix A, where the importance of properly designing certain system parameters to ensure the orthogonality is also demonstrated. Compared to the one-dimensional SRS, the DD-SRS relaxes the orthogonality conditions for multiple users by introducing an additional dimension and enlarges the capacity of multiple access. By performing a 2D-FFT operation [22], i.e., an IFFT operation in the delay domain and an FFT operation in the Doppler domain, an RD map can be obtained for each user or at the BS. As illustrated in Figure 4a, when the assumptions hold true, the modulation in the DD domain on the SRS enables perfect separation of SEI of different users. It is evident that, compared to one-dimensional SRS, DD-SRS supports orthogonal access for a greater number of users, potentially increasing the multi-user capacity. The orthogonality of the SRS can be maintained easily in traditional LTE systems since the SRS transmission is managed by the BS. However, the orthogonality can be broken due to increased SRS bursts and potential resource collisions caused by more frequent and complex sensing requirements in the V2X ISAC system. Additionally, as illustrated in Figure 4b, the high mobility of users could disrupt pilot orthogonality, causing pilot contamination in the DD domain and thus the strong IUI in the RD maps. Moreover, the wide dynamic range of the delay and Doppler shifts in the V2X channels makes maintaining user orthogonality with non-adaptive phase shifts more difficult. To address these issues, we propose a DL scheme based on pixel segmentation at ISAC vehicles or BSs to mitigate the IUI SEI terms in ${\hat{H}}_{k}$.

It is noted that the proposed DD-SRS framework can be compatible with existing 4/5G systems with some necessary modifications. For example, the DD-SRS framework can be implemented within the existing aperiodic SRS transmission triggered by BSs [27] for on-demand and high-precision sounding needs. Since the existing SRS transmission considering user multiplexing involves the BS allocating SRS sequences with different cyclic shifts, some modifications can be introduced into the phase shift configurations and the corresponding RRC protocol for the additional Doppler domain based on the existing mechanism. As for the user equipment (UE), it also requires software updates to generate the DD-SRS with user-specific offsets. Simultaneously, the receiver at the BS or the UE employs enhanced 2D matched filtering techniques for the CSI recovery. Considering the orthogonality contamination, the receiver may incorporate certain interference mitigation algorithms, such as the one based on image processing proposed in the following section. Therefore, increased computational capacity and efficiency based on updated software and hardware platforms for 2D processing may be necessary.

### 3.2. Interference Mitigation Scheme Based on Image-Pixel-Segmentation-Based Neural Network

With the interfered SEI ${\hat{H}}_{k}$ in (10), we first obtain the RD map ${\hat{Z}}_{k}$ in the DD domain through the 2D-FFT operation. With non-orthogonal multiple access, ${\hat{Z}}_{k}$ is given by ${\hat{Z}}_{k}=Z_{k}+{\tilde{Z}}_{k}+{\tilde{Z}}_{W,k}$, where $Z_{k},{\tilde{Z}}_{k},{\tilde{Z}}_{W,k}$ denotes the RD map obtained by performing a 2D-FFT operation over the local SEI $H_{k}$, the IUI SEI ${\tilde{H}}_{k}$, and the noise term ${\tilde{W}}_{k}$, respectively. To recover the local SEI $H_{k}$ from ${\hat{Z}}_{k}$, we then model the interference cancellation problem of multiple users as a two-label classification task and feed ${\hat{Z}}_{k}$ into a trained image segmentation network. Specifically, the first label is obtained with $Z_{k}$ and the second label is obtained with ${\tilde{Z}}_{k}$. Additionally, the classification task is practically approached as two separate binary classification problems since some pixels may belong to both classes contemporarily. With the predicted probabilities of the network, we obtain a binary mask ${\hat{U}}_{k,1}$ which identifies the pixels belonging to the first label based on a probability threshold $p_{\mathrm{th}}$. Next, the estimated local SEI in the DD domain is given by ${\hat{Z}}_{k}^{\prime }={\hat{Z}}_{k}⊙{\hat{U}}_{k,1}$. ${\hat{Z}}_{k}^{\prime }$ directly provides the intuitive information of the sensing environment in the DD domain. Finally, through executing an inverse transformation of the 2D-FFT operation on ${\hat{Z}}_{k}^{\prime }$, we can recover the local SEI $H_{k}$ for the *k*-th user.

**Figure 5:**
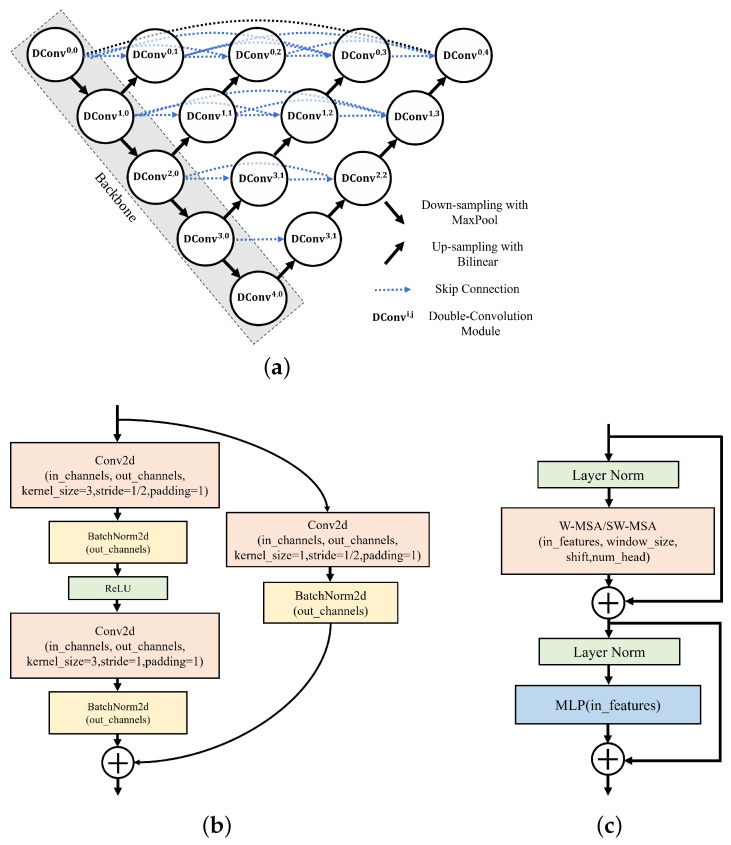
The model architecture: (a) the UNetPP architecture; (b) the RSB-based architecture; (c) the STB-based architecture.

The basic network structure we employ for image segmentation tasks on the RD maps is the four-layer UNet++ (UNetPP) architecture introduced in [28], as illustrated in Figure 5a. UNetPP is an enhanced CNN architecture and an extended version of the classical UNet structure. It is characterized by nested and dense skip connections between the dual-convolution blocks (DCBs, denoted by ${\mathrm{Dconv}}^{i,j}$ in Figure 5a), which are designed to improve feature reuse and multi-scale feature fusion capabilities. The key distinction of UNetPP from the traditional UNet architecture lies in its densely connected skip pathways in the decoder path. These pathways, built upon the original skip connections in the UNet, incorporate additional intermediate convolutional modules, enabling the fusion of features from various levels. With the dense skip connections, the network conducts multiple combinations and processes at different resolutions, capturing greater contextual information and fine details, and thus enhancing the boundary and detail handling in segmentation tasks and improving the segmentation accuracy.

As shown in Figure 5a, in our proposed models, the input size of the RD map is denoted by ${\mathrm{FMP}}_{0}=\left[C_{0},H_{0},W_{0}\right]$ where $C_{0},H_{0},W_{0}\geq 1$ is the number of channels and the number of pixels in the horizontal and vertical dimensions, respectively. ${\mathrm{FMP}}_{q}=\left[C_{q},H_{q},W_{q}\right]$ denotes the size of the feature map after the *q*-th encoding stage.

In our work, we utilize different encoder blocks in the backbone of the UNetPP-architecture-based network. In addition to the original DCBs utilized in [28,29], we investigate exploiting the residual blocks (RSBs) in [30] and the swin-transformer blocks (STBs) in [31] as the backbone in the UNetPP-based network to enhance the performance, and the two models are named Swin-UNetPP and UNetPP-Res34, respectively. The RSB-based and STB-based architectures are illustrated in Figure 5b,c. It is worth noting that the backbone based on the STBs in the model Swin-UNetPP is different from the one based on the DCBs and the RSBs in the model UNetPP and UNetPP-Res34. The latter two models rely on convolutional operations and pooling layers (e.g., max pooling) to progressively extract image features and reduce the spatial dimensions of feature maps in the backbone. In contrast, Swin-UNetPP employs patch embedding and patch merging for downsampling in the backbone. In the first encoding stage, Swin-UNetPP divides the input RD map into fixed-size patches, with each patch being embedded into a high-dimensional feature space through a linear transformation, a process known as patch embedding. This approach transforms each local region of the RD map into a vector representation, facilitating subsequent processing by the transformer block. For downsampling, the model uses a patch merging operation, which reduces the spatial dimensions of the feature map by merging multiple adjacent patches, rather than relying on traditional pooling operations. Therefore, ${\mathrm{FMP}}_{1}$ of Swin-UNetPP is given by ${\mathrm{FMP}}_{1}=\left[C_{0},H_{0}/h_{p},W_{0}/w_{p}\right]$, where $\left[h_{p},w_{p}\right]$ is the patch size in the patch embedding operation.

Our loss function is formulated as a weighted sum of Binary Cross-Entropy (BCE) and Dice Loss. Dice Loss is a specialized loss function for image segmentation tasks, particularly effective in addressing class imbalance issues [32,33]. The calculation formula for the Dice Loss of a given class *c* is as follows:

(12) $$L_{\mathrm{Dice},c}=1-\frac{2\sum_{i=1}^{I}p_{i}t_{i}}{\sum_{i=1}^{I}p_{i}^{2}+\sum_{i=1}^{I}t_{i}^{2}},$$

where $p_{i}$ is the predicted probability and $t_{i}\in \left\{0,1\right\}$ is the ground truth of the pixel *i*. When the pixel *i* belongs to class *c*, $t_{i}=1$. I is the total number of pixels of the image. The BCE loss is given by

(13) $$L_{\mathrm{BCE},c}=-\frac{1}{I}\sum_{i=1}^{I}\left[t_{i}\log\left(p_{i}\right)+\left(1-t_{i}\right)\log\left(1-p_{i}\right)\right].$$

By performing a weighted summation of these two loss functions and averaging over both classes, we obtain the formulation for the weighted sum loss function as follows:

(14) $$L_{\mathrm{total}}=\frac{1}{2}\sum_{c=1}^{2}\left[\alpha \times L_{\mathrm{Dice},c}+\beta \times L_{\mathrm{BCE},c}\right].$$

## 4. Simulation and Results

### 4.1. Configuration

Based on the deterministic channel model considered in Section 2, we utilize MATLAB R2023b to generate a substantial amount of channel data. We randomly generate 2D coordinates and 2D velocity vectors based on a uniform distribution to simulate a complex vehicular network wireless transmission environment. Additionally, the distances and velocities of multiple users are generated within the range $\left[d_{\min},d_{\max}\right]$ and $\left[v_{\min},v_{\max}\right]$, respectively. The radar cross-section (RCS) and the received SNR settings for the received signals are referenced from [17]. The range of the RCS for stationary clutter is between 5 and 20 dBsm, while the target’s RCS ranges from 15 to 25 dBsm. The amplitudes of the direct path are modeled using the Friis transmission equation [34], whereas the reflective paths are modeled using the radar range equation [35], with phases being randomly generated with a uniform distribution. In a single Monte Carlo simulation step, after randomly generating the channels and DD-SRS sequences as in (9) for *K* users, we obtained the RD maps of size [H,W] for *K* users, ${\hat{Z}}_{k},k\in K$, following the received signal models and processing steps introduced in Section 2 and Section 3. For each scenario, we use 5000 realizations of RD maps as training data, 2000 realizations as validation data, and 1000 as test data.

For the generation of two labels, we use an amplitude threshold $\alpha_{\mathrm{th}}$ to generate a binary-value label mask $U_{j,c}$ of size $\left[H,W\right]$ for the *c*-th label of the *j*-th RD map sample. The pixels whose values higher than $\alpha_{\mathrm{th}}$ in the RD map are marked as 1, and the pixels whose values are lower than $\alpha_{\mathrm{th}}$ are marked as 0 in the generated label map. As for the training process, we employ the SGD optimizer for both networks, with an initial learning rate of 0.08 and a weight decay rate of 0.0001. After obtaining the network’s predicted probability outputs of all pixels from the sigmoid layer, we use a probability threshold $p_{\mathrm{th}}=0.5$ to determine whether a pixel belongs to a label and then obtain the binary mask ${\hat{U}}_{j,c}$ for the *j*-th RD map sample.

The simulation parameters considered are listed in Table 1. The maximum unambiguous range for vehicular sensing is given by $R_{\max}=312$ m, and the maximum Doppler shift available for multiple access based on the DD-SRS can reach up to ±60 kHz. We consider utilizing 10% of this range, namely ±6 kHz, to preliminarily validate the feasibility of our proposed ISAC V2X framework and the interference cancellation scheme. The scenario considered is the typical urban scenario where very-high-speed vehicles (e.g., high-speed trains) and flying objects (e.g., Unmanned Aerial Vehicles) are not considered. Additionally, the parameters of the OFDM system influence the system performance. For example, the subcarrier spacing Δf considered is designed to be several times the maximum Doppler frequency shift to ensure that the inter-subcarrier interference can be neglected [22]. Meanwhile, the bandwidth and the size of 2D FFT operations are chosen to be large enough to ensure the resolution of the RD maps to recover the local SEI. Furthermore, the trade-off between the processing latency and the performance should be considered in practical implementation since some V2X services require ultra-low latency. In our simulations, we use the network UNet-Res34, which lacks multi-layer dense connections in the decoder, as the baseline for the network UNetPP, SwinUNetPP and UNetPP-Res34 where the decoder is built upon the UNetPP-architecture-based decoder. The performance of different models on the segmentation task for the *j*-th RD map sample is measured by the mean Intersection over Union (mIoU) of two labels, which is given by

(15) $${\mathrm{mIoU}}_{j}=\frac{1}{2}\sum_{c=1}^{2}\frac{\sum_{i=1}^{I}{\hat{u}}_{c,i}u_{c,i}}{\sum_{i=1}^{I}{\hat{u}}_{c,i}+\sum_{i=1}^{I}u_{c,i}},$$

where *I* is the total number of pixels, and ${\hat{u}}_{c,i},u_{c,i}$ is the *i*-th element of the binary mask ${\hat{U}}_{j,c},U_{j,c}$. The performance metric for a model is the average mIoU over *J* samples in the dataset. With the output binary mask ${\hat{U}}_{j,1}$, we can recover the local SEI $H_{j}$. We also measure the performance of our proposed algorithm with the normalized mean square error (NMSE) between $H_{j}$ and ${\hat{H}}_{j}$, which is given by

(16) $${\mathrm{NMSE}}_{j}=\frac{{\left|\left|H_{j}-{\hat{H}}_{j}\right|\right|}^{2}}{{\left|\left|H_{j}\right|\right|}^{2}}.$$

### 4.2. mIoU Performance of Proposed Framework and Scheme

**Figure 6:**
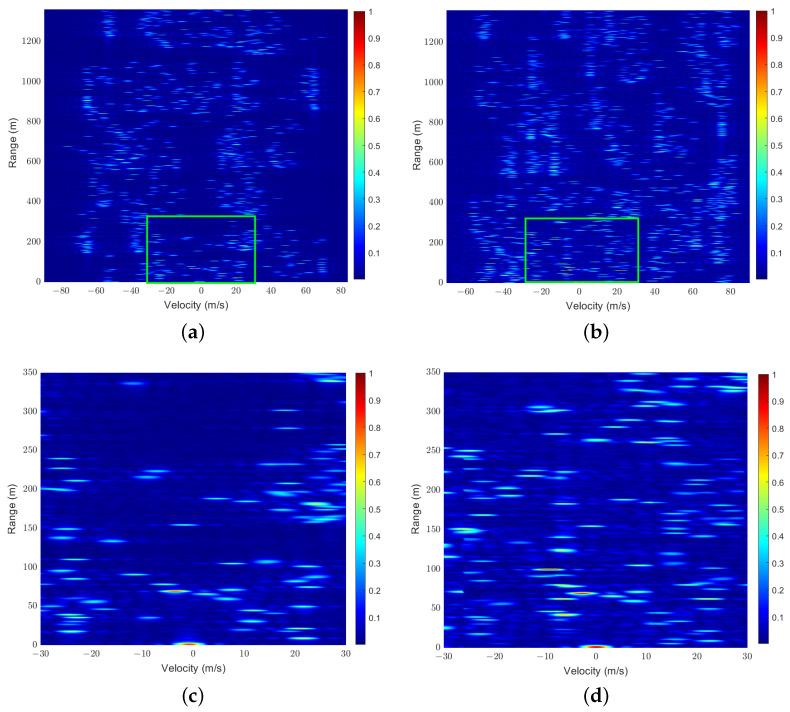
RD Map samples: (a) K=25; (b)K=36; (c) the zoomed-in version with K=25; (d) the zoomed-in version with K=36.

We first demonstrate in Figure 6a,b a sample of RD maps, $\hat{Z}$, generated for scenarios with K=25 and K=36 at a signal-to-noise power ratio (SNR) of 15 dB, where the local SEI of the user is represented by the region enclosed within the green rectangular box. Additionally, the zoomed-in version of the parts within the green rectangular box is shown in Figure 6c,d. It can be observed that when the number of users is relatively small, the channels can essentially remain orthogonal, allowing the local SEI of each user to be easily separated. However, in more congested user scenarios, maintaining channel orthogonality becomes challenging, and signals from other users act as interference, making it difficult for direct separation methods to accurately extract the local SEI.

**Figure 7:**
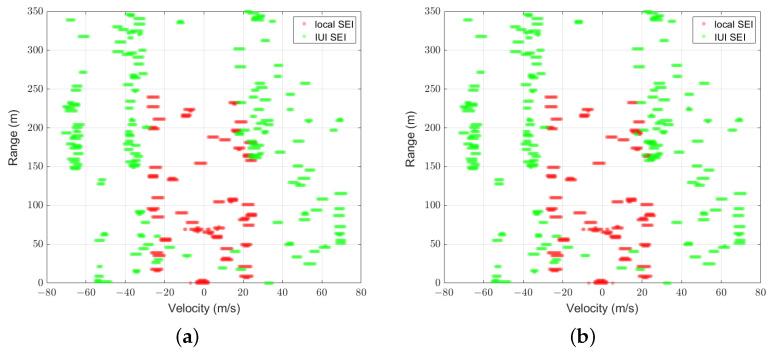
Results with K=25,mIoU=0.89: (a) binary label mask; (b) predicted mask of UNetPP-Res34.

**Figure 8:**
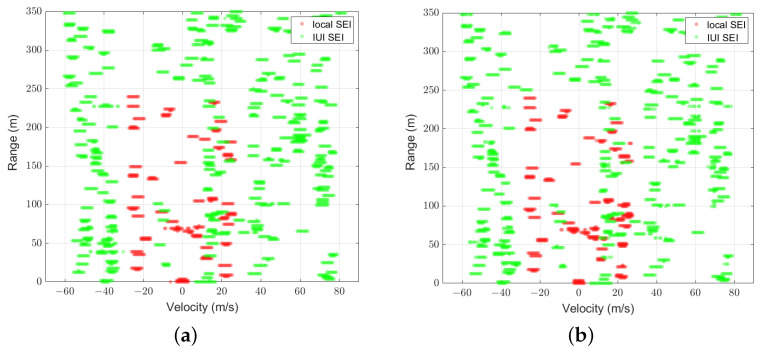
Results with K=36,mIoU=0.84: (a) binary label mask; (b) predicted mask of UNetPP-Res34.

Figure 7 and Figure 8 illustrate the comparison between the predicted mask $\hat{U}$ of UNetPP-Res34 and the label masks U for scenarios with 25 and 30 users at an SNR of 15 dB, respectively. Specifically, Figure 7a and Figure 8a demonstrate the zoomed-in areas of the corresponding label masks U, where the red dots represent the pixels corresponding to the local SEI H, and the green dots represent the pixels corresponding to the IUI SEI. It can be observed that though the IUI is higher with the condition of more users, the SEI segmentation network based on UNetPP achieves commendable mIoU performance.

**Figure 9:**
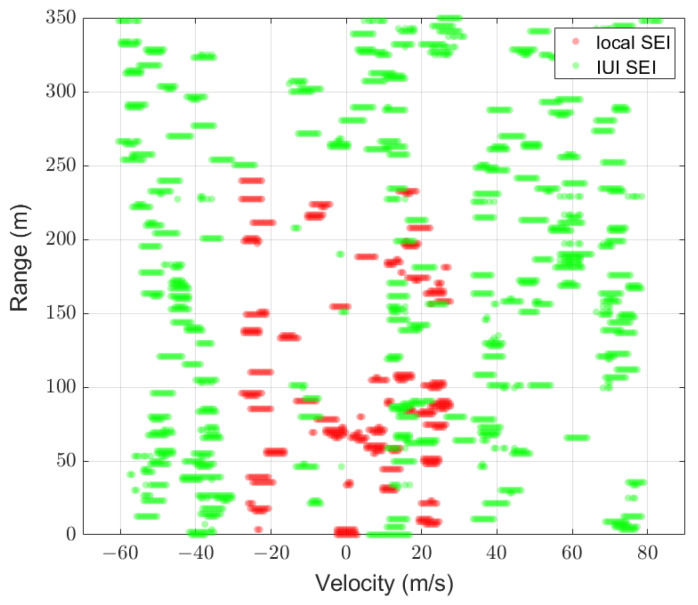
Predicted mask of UNetPP-Res34 with K=36,mIoU=0.81.

Figure 9 shows the predicted mask for the scenario with 36 users at a reduced SNR of 5 dB compared to Figure 8. It is shown that as the SNR decreases, the segmentation network exhibits poorer mIoU performance. Specifically, compared to the result in the scenario with a higher SNR in Figure 8b, a greater number of isolated pixels corresponding to the noise are predicted as positive samples belonging to the first and second labels.

Apart from the level of the IUI and the SNR, the performance of the proposed scheme is also influenced by the sizes of the feature maps in the models. Table 2 presents the performance of models on the test dataset with different sizes of the feature maps. In our work, ${\mathrm{FMP}}_{q}$ of the last four encoding stages satisfies

(17) $${\mathrm{FMP}}_{q}=\left[C_{q-1}*2,H_{q-1}/2,W_{q-1}/2\right],q=\left\{2,...,5\right\}.$$

We vary ${\mathrm{FMP}}_{1}$ to change the sizes of feature maps in the proposed models. While $\left[H_{1},W_{1}\right]$ in the UNetPP and UNetPP-Res34 can be designed to be equal to the original input size, the original input size is too large for the transformer block to process. Therefore, we fix the patch size as [2,2] in the patch embedding operation in the first encoding stage for Swin-UNetPP, resulting in the fixed $\left[H_{1},W_{1}\right]$ as [384,144]. Additionally, the number of STBs in the other four encoding stages is [2,2,8,2], with attention head counts of $\left(3,6,12,24\right)$ and a window size of 7 for the windowed self-attention mechanism. In UNetPP, the parameters in the blocks ${\mathrm{DConv}}^{i,j}$ are fixed as kernelsize=3,stride=1,padding=1. As for UNetPP-Res34, we substitute the encoding stage based on the DCBs in UNetPP with the layers of the same number of input and output channels in ResNet34.

It can be observed from Table 2 that increasing the size of the feature maps can enhance segmentation performance due to more information learned by the models from the feature maps at the cost of higher computational complexity. Specifically, retaining more pixel information in the first stage significantly improves the mIoU performance. UNetPP and UNetPP-Res34 exhibit the best performance at an SNR of 15 dB when ${\mathrm{FMP}}_{1}=\left[16,768,288\right]$, achieving the mIoU values of approximately 0.85 and 0.87, respectively. This suggests that, for the data and segmentation tasks considered, downsampling easily leads to the loss of spatial information learned by the model.

Moreover, UNetPP-Res34 improves the mIoU performance by approximately 2.5% compared to UNetPP. This performance gain is attributed to both the deeper architecture of the backbone and the residual connections in the RSBs. On one hand, the numbers of RSBs in the second, third and fourth encoding stages of UNetPP-Res34 are set as 3,4,6, respectively, which are greater than the numbers of DCBs in the encoding stages of UNetPP, therefore providing a larger receptive field. On the other hand, incorporating residual connections in the RSBs allows more effective propagation of the feature maps, enabling the network to more "aggressively" learn the information of small targets and update the weights more efficiently. This is especially effective since the number of positive labels is extremely small in our scenarios compared to the number of negative labels, i.e., the background information in the RD map, resulting in the numerator and denominator of the Dice Loss being close to zero simultaneously. Therefore, the gradient information during training steps becomes very weak sometimes and the network struggles to effectively learn the information and improve the performance. Even the BCE loss is considered in our loss function.

As for SwinUNetPP, it is also observed that increasing the number of feature channels can improve performance to a certain extent. Despite the loss of some spatial details due to downsampling, networks with a transformer architecture leverage the large receptive field introduced by the window attention mechanism. Consequently, they also achieve good performance with ${\mathrm{FMP}}_{1}=\left[16,384,144\right]$, reaching an mIoU of 0.8561 at an SNR of 15dB. It can be also observed that when the model’s forward pass size is approximately similar, the segmentation performance of Swin-UnetPP is inferior to that of UnetPP-Res34. This could be due to the fact that the STB-based model relies on window-based self-attention to tackle the large input data of size [768,288], and therefore potentially loses the ability to capture and learn comprehensive channel information.

### 4.3. NMSE Performance of Proposed Framework and Scheme

**Figure 10:**
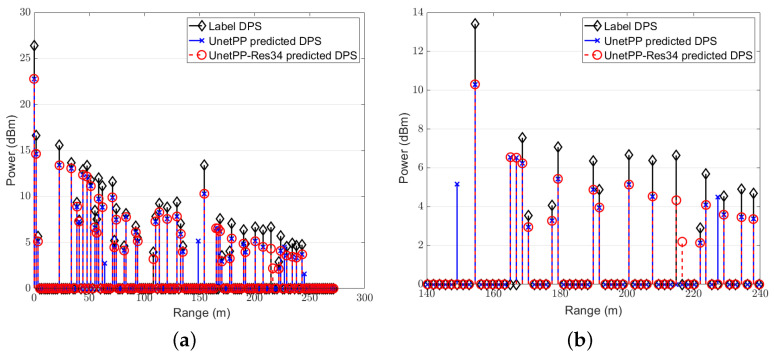
RPS with K=25: (a) the full diagram; (b) the zoomed-in diagram.

In this subsection, we focus on the comparison between the NMSE performance of different image segmentation models. We first demonstrate the estimated range power spectrum (RPS) diagrams obtained through segmentation by the network UNetPP and UNetPP-Res34 with 25 users at an SNR of 15dB in Figure 10 since we usually put more emphasis on the exact locations of surrounding clutter and objects in realistic ISAC V2X scenarios. Specifically, Figure 10b provides a zoomed-in view of Figure 10a. The amplitude threshold $\alpha_{\mathrm{th}}$ for label generation is set at 0.1 here, with the corresponding energy threshold being −20 dB. It can be observed that the segmentation model UNetPP-Res34, which combines the UNetPP-based CNN with residual connections, facilitates a more precise perception of the surrounding environment compared to the standard UNetPP network.

**Figure 11:**
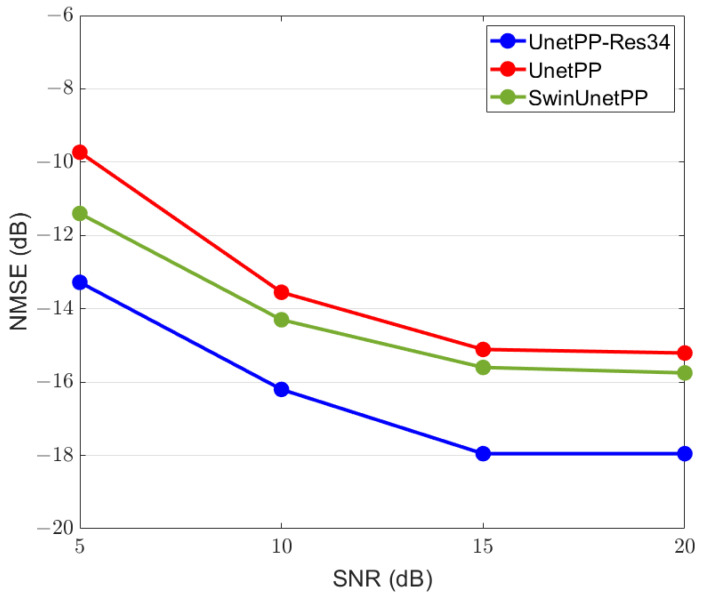
Average NMSE of local SEI $H_{k}$ with K=25 versus SNR with different models.

Figure 11 illustrates the NMSE performance curves for class 1 under different SNR conditions for the test dataset, following image segmentation based on random access and frequency-domain digital filtering in a scenario with 25 users. It is observed that as the SNR increases, the average NMSE decreases. This is because when the noise power is comparable to signal power, more noise is likely to be mistaken as part of the local SEI. Additionally, the superimposed noise can also lead to attenuation of the useful signal amplitude, and thereby can impact the network’s segmentation capabilities.

Table 3 presents a comparative analysis of the results obtained from UnetPP-Res34 and the standard UnetPP across scenarios with different numbers of users. It is observed that the advantages of residual connections become more pronounced under conditions of low SNR and expanded capacity of multiple access.

## 5. Conclusions

In this paper, we introduced an innovative multi-user ISAC framework based on the DD-SRS to tackle the challenges of multiple access in ISAC-enabled V2X networks under limited TF resources and severe IUI. By maximizing the utilization of SRS sequences’ bandwidth and local sensing capabilities of ISAC users, the framework enhances the efficiency of resource usage, enabling more users to share the same TF resources and ensuring robust multi-user coexistence. Moreover, we proposed a pixel-segmentation- and DL-based approach to minimize the IUI in non-orthogonal ISAC scenarios, leveraging RD maps for improved performance. The UNetPP-Res34 model, which integrates residual connections with dense skip connections in the encoder–decoder stages, achieved commendable mIoU performance across various SNRs and feature map sizes. Although increasing the feature map sizes could enhance the performance by allowing the network to learn more information, it could also increase the memory and time required for network inference. The model’s ability to effectively learn and retain detailed localized information under diverse SNR conditions highlighted its practical application potential for enhancing the multi-access capacity in resource-restricted scenarios. Additionally, the NMSE performance showed that UNetPP-Res34 consistently outperforms the standard UNetPP model in various user scenarios and SNR conditions, underscoring the advantages of residual connections. This paper primarily focuses on the interference cancellation in ISAC V2X systems utilizing random multiple access based on the DD-SRS. In future research, we will explore optimizing the fixed SRS and corresponding DL-based interference mitigation algorithms for BSs to maximize multiple-access capacity under diverse conditions regarding the vehicular mobility. Furthermore, we will investigate the trade-off between real-time computational complexity and performances of the DL-based methods.

## Figures and Tables

**Table 1:** Simulation parameters.

Number of subcarriers	N=768
Number of symbols	M=256
Carrier frequency	$f_{c}=6$ GHz
Subcarrier spacing	Δf=120 kHz
Total bandwidth	B=102 MHz
OFDM effective symbol duration	$T_{s}=8.3$ µs
OFDM CP duration	$T_{CP}=2$ µs
OFDM symbol duration	$T_{sym}=10.3$ µs
Size of the RD Map	$\left[H_{0},W_{0}\right]=\left[768,288\right]$
Number of users	K=25
$\left[d_{\min},d_{\max}\right]$	[5,300] m
$\left[v_{\min},v_{\max}\right]$	[2,25] m/s

**Table 2:** mIoU with different sizes of feature maps with K=25.

Models	${\mathrm{FMP}}_{1}$	SNR = 5 dB	SNR = 15 dB
UNet-Res34	[8,768,288]	0.732	0.7487
	[16,384,144]	0.744	0.771
	[16,768,288]	0.75	0.783
	[32,768,288]	0.753	0.787
UNetPP	[8,768,288]	0.8136	0.8415
	[16,384,144]	0.7794	0.8047
	[16,768,288]	0.8268	0.8518
	[32,768,288]	0.8269	0.8475
SwinUNetPP	[8,384,144]	0.8025	0.8298
	[16,384,144]	0.8335	0.8561
	[32,384,144]	0.837	0.8592
UNetPP-Res34	[8,768,288]	0.8422	0.8691
	[16,384,144]	0.8276	0.8403
	[16,768,288]	0.851	0.8764
	[32,768,288]	0.8504	0.8716

**Table 3:** NMSE with different models based on the UNetPP architecture with the unit of dB.

Models	*K*	SNR = 5 dB	SNR = 10 dB	SNR = 20 dB
UNetPP-Res34	25	−13.278	−16.2	−17.954
	36	−8.18047	−11.2551	−13.4811
UNetPP	25	−9.73	−13.55	−15.207
	36	−3.6609	−7.7904	−9.7965

**Figure A1:**
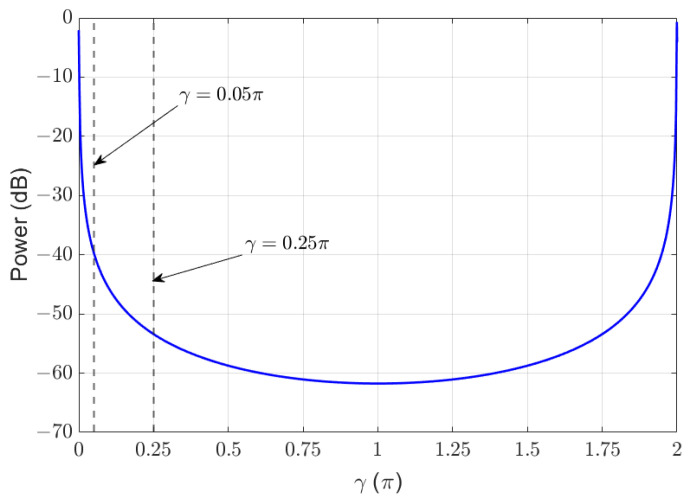
${\left|f\left(\gamma \right)\right|}^{2}$ when N=768,Δf=120 kHz.

## Data Availability

The data presented in this study are available on request from the corresponding author.

## Appendix A. Analysis of the Condition of Orthogonality for the DD-SRS in (11)

In this appendix, we first employ the continuous Fourier Transform (FT) to analyze the condition of orthogonality in (11) based on the procedures introduced in Section 3.2. Then, we move on to discuss how the orthogonality can be ensured with DFT operations considering the parameter design in practical implementation of the DD-SRS.

### Appendix A.1. Orthogonality with FT Operations

Based on the estimated local SEI with the DD-SRS in (10), we have that

(A1) $${\hat{H}}_{k}=H_{k}+{\tilde{H}}_{k}+{\tilde{W}}_{k}$$

where

(A2) $$H_{k}=\sum_{l=0}^{L_{k}-1}\alpha_{k,l}b\left(\tau_{k,l}\right)c^{H}\left(\nu_{k,l}\right),$$

(A3) $${\tilde{H}}_{k}=\sum_{i=0,i\neq k}^{K}\sum_{l=0}^{L_{i}-1}\alpha_{i,l}b\left(\tau_{i,l}+\Delta_{ik}\right)c^{H}\left(\nu_{i,l}+\delta_{ik}\right),$$

(A4) $$\begin{array}{cc} \; & b\left(\tau \right)={\left[\begin{array}{c} 1,e^{-j2\pi \Delta f\tau },\ldots ,e^{-j2\pi (N-1)\Delta f\tau } \end{array}\right]}^{T},\\ \; & c\left(\nu \right)={\left[\begin{array}{c} 1,e^{-j2\pi \nu T_{\mathrm{sym}}},\ldots ,e^{-j2\pi \left(M-1\right)\nu T_{\mathrm{sym}}} \end{array}\right]}^{T}. \end{array}$$

As described in Section 3.2, to estimate $H_{k}$ from ${\hat{H}}_{k}$ based on the DD-SRS, we firstly apply a 2D FFT operation to ${\hat{H}}_{k}$ to obtain its representation in the DD domain, and then use a digital low-pass filter (LPF) matrix which serves as a mask and adopt the IFFT operation to recover $H_{k}$.

Denoting the FT operation as $F_{1}$ and the 2D FT operation as $F_{2}$, we have that

(A5) $$F_{2}\left\{{\hat{H}}_{k}\right\}=F_{2}\left\{H_{k}\right\}+F_{2}\left\{{\tilde{H}}_{k}\right\}+F_{2}\left\{W_{k}\right\}.$$

Then, the LPF function $G_{\left(\tau ,\nu \right)}$ given in (A6) in the DD domain is adopted to $F_{2}\left\{{\hat{H}}_{k}\right\}$, where $g_{\tau },g_{\nu }>0$ is the designed guard interval of the filter in the delay and Doppler domains, respectively.

(A6) $$G_{\left(\tau ,\nu \right)}=\left\{\begin{array}{cc} 1, & \left|\tau \right|\leq \tau_{k,\max}+g_{\tau }\;\mathrm{and}\;\left|\nu \right|\leq \nu_{k,\max}+g_{\nu }\\ 0, & \mathrm{otherwise} \end{array}\right.,$$

The filtered signal is thereby given by

(A7) $$F_{2}\left\{{\hat{H}}_{k}\right\}\cdot G_{\left(\tau ,\nu \right)}=F_{2}\left\{H_{k}\right\}+F_{2}\left\{{\tilde{H}}_{k}\right\}\cdot G_{\left(\tau ,\nu \right)}+F_{2}\left\{W_{k}\right\}\cdot G_{\left(\tau ,\nu \right)}.$$

In the following steps, we focus on proving that the condition in (11) ensures that the IUI-SEI component $F_{2}\left\{{\tilde{H}}_{k}\right\}\cdot G_{\left(\tau ,\nu \right)}$ can be eliminated.

It is noted that b,c defined in (5) represents a sampled complex exponential function with a fundamental frequency of $\Delta f,T_{\mathrm{sym}}$, respectively. Therefore, $b\left(\tau_{i,l}+\Delta_{ik}\right),c^{H}\left(\nu_{i,l}+\delta_{i,k}\right)$ is a delayed version of b(τ),c(ν), respectively, and it follows that

(A8) $$\begin{array}{c} F_{1}{\left\{b\left(\tau_{i,l}+\Delta_{ik}\right)\right\}}_{\tau }=\delta_{D}\left(\tau -\left(\tau_{i,l}+\Delta_{ik}\right)\right),\\ F_{1}{\left\{c^{H}\left(\nu_{i,l}+\delta_{i,k}\right)\right\}}_{\nu }=\delta_{D}\left(\nu -\left(\nu_{i,l}+\delta_{i,k}\right)\right), \end{array}$$

where $\delta_{D}\left(\cdot \right)$ is the Dirac delta function. Then, the FT of the product of the two vectors is

(A9) $$\begin{array}{cc} \; & F_{2}{\left\{b\left(\tau_{i,l}+\Delta_{ik}\right)c^{H}\left(v_{i,l}+\delta_{i,k}\right)\right\}}_{\tau ,\nu }\\ \; & =F_{2}{\left\{b\left(\tau_{i,l}+\Delta_{ik}\right)\right\}}_{\tau }*F_{2}{\left\{c^{H}\left(\nu_{i,l}+\delta_{i,k}\right)\right\}}_{\nu }\\ \; & =\delta_{D}\left(\tau -\left(\tau_{i,l}+\Delta_{ik}\right)\right)\delta_{D}\left(\nu -\left(\nu_{i,l}+\delta_{i,k}\right)\right). \end{array}$$

Therefore, $F_{2}{\left\{{\tilde{H}}_{k}\right\}}_{\left(\tau ,\nu \right)}$ is given by

(A10) $$\begin{array}{c} F_{2}{\left\{{\tilde{H}}_{k}\right\}}_{\left(\tau ,\nu \right)}=\sum_{i=0,i\neq k}^{K}\sum_{l=0}^{L_{i}-1}\alpha_{i,l}\delta \left(\tau -\left(\tau_{i,l}+\Delta_{ik}\right)\right)\delta \left(\nu -\left(\nu_{i,l}+\delta_{i,k}\right)\right). \end{array}$$

When $\Delta_{ik}>\tau_{k,\max}$ or $\delta_{i,k}>\nu_{k,\max}+\nu_{i,\max}$ holds with ∀i∈K,i≠k, it follows that

(A11) $$\tau_{i,l}+\Delta_{ik}>\tau_{i,l}+\tau_{k,\max}\geq \tau_{k,\max},$$

or

(A12) $$\begin{array}{cc} |\nu_{i,l}+\delta_{i,k}| & \geq \;|\delta_{i,k}|\;-\;|\nu_{i,l}|\;>\left(\nu_{k,\max}+\nu_{i,\max}\right)-\nu_{i,\max}=\nu_{k,\max}. \end{array}$$

Combining (A11) and (A12), it can be observed that the following equation holds for ∀i∈K,i≠k when the condition in (11) is satisfied

(A13) $$\delta \left(\tau -\left(\tau_{i,l}+\Delta_{ik}\right)\right)\delta \left(\nu -\left(\nu_{i,l}+\delta_{i,k}\right)\right)=0.$$

Therefore, $F_{2}{\left\{{\tilde{H}}_{k}\right\}}_{\left(\tau ,\nu \right)}$ is zero within the interested region $\left|\tau \right|\leq \tau_{k,\max}+g_{\tau },\left|\nu \right|\leq \nu_{k,\max}+g_{\nu }$, indicating that the term $F_{2}\left\{{\tilde{H}}_{k}\right\}\cdot G_{\left(\tau ,\nu \right)}$ corresponding to the IUI SEI can be eliminated.

Finally, we can apply the inverse 2D FT to obtain the estimated $H_{k}$ as

(A14) $$H_{k}^{\mathrm{est}}=F^{-1}\left\{F_{2}\left\{{\hat{H}}_{k}\right\}\cdot G_{\left(\tau ,\nu \right)}\right\}\approx H_{k}+W_{k}^{\mathrm{filtered}},$$

where the remaining noise term $W_{k}^{\mathrm{filtered}}$ is the filtered version of the original noise, which can be further mitigated through additional signal processing techniques.

### Appendix A.2. Orthogonality with DFT Operations

With DFT operations in practical implementation, the orthogonality is influenced since the ideal Dirac delta function is not achievable. In the following steps, we further prove that the IUI-SEI component can be eliminated given the orthogonality condition in (11) and the signal models in (A1)–(A4) with a few parameters properly designed.

Let us denote the 2D DFT operation with the same size as the input matrix by ${\tilde{F}}_{2}\left\{\cdot \right\}$; then, it is obtained that

(A15) $${\tilde{F}}_{2}\left\{{\hat{H}}_{k}\right\}={\tilde{F}}_{2}\left\{H_{k}\right\}+{\tilde{F}}_{2}\left\{{\tilde{H}}_{k}\right\}+{\tilde{F}}_{2}\left\{W_{k}\right\},$$

where the local SEI and the IUI-SEI components are given by

(A16) $$\begin{array}{cc} \; & {\tilde{F}}_{2}{\left\{H_{k}\right\}}_{p,q+\frac{M}{2}}=\frac{1}{NM}\sum_{l=0}^{L_{k}-1}\alpha_{k,l}\cdot \sum_{n=0}^{N-1}\sum_{m=M}^{M-1}b_{n}\left(\tau_{k,l}\right)c_{m}^{*}\left(\nu_{k,l}\right)e^{j2\pi (\frac{pn}{N}-\frac{qm}{M})},\\ \; & {\tilde{F}}_{2}{\left\{{\tilde{H}}_{k}\right\}}_{p,q+\frac{M}{2}}=\frac{1}{NM}\sum_{i=0,i\neq k}^{K}\sum_{l=0}^{L_{i}-1}\alpha_{i,l}\cdot \sum_{n=0}^{N-1}\sum_{m=0}^{M-1}b_{n}\left(\tau_{i,l}+\Delta_{ik}\right)c_{m}^{*}\left(\nu_{i,l}+\delta_{ik}\right)e^{j2\pi (\frac{pn}{N}-\frac{qm}{M})},\\ \; & \forall p\in \{0,1...,N-1\},q\in \{-M/2,\ldots ,0,\ldots ,M/2-1\}, \end{array}$$

where ${\tilde{F}}_{2}{\left\{H_{k}\right\}}_{p,q+\frac{M}{2}}$ denotes the $\left[p,q+\frac{M}{2}\right]$-th element of ${\tilde{F}}_{2}\left\{H_{k}\right\}$, and $b_{n}\left(\tau \right),c_{m}\left(\nu \right)$ are the *n*-th and *m*-th elements of b(τ) and c(ν), respectively. Substituting the definitions of b(τ) and c(ν) in (A4) into (A16), it follows that

(A17) $$\begin{array}{cc} \; & {\tilde{F}}_{2}{\left\{H_{k}\right\}}_{p,q+\frac{M}{2}}=\frac{1}{NM}\sum_{l=0}^{L_{k}-1}\alpha_{k,l}\cdot \sum_{n=0}^{N-1}e^{-j\gamma_{k,l}^{p}n}\sum_{m=0}^{M-1}e^{j\gamma_{k,l}^{q}m},\\ \; & {\tilde{F}}_{2}{\left\{{\tilde{H}}_{k}\right\}}_{p,q+\frac{M}{2}}=\frac{1}{NM}\sum_{i=0,i\neq k}^{K}\sum_{l=0}^{L_{i}-1}\alpha_{i,l}\cdot \sum_{n=0}^{N-1}e^{-j\gamma_{i,l}^{p}n}\sum_{m=0}^{M-1}e^{j\gamma_{i,l}^{q}m}, \end{array}$$

where $\gamma_{i,l}^{p}≜2\pi \left(\Delta f\left(\tau_{i,l}+\Delta_{i,k}\right)-p/N\right),\gamma_{i,l}^{q}≜2\pi \left(T_{\mathrm{sym}}\left(\nu_{i,l}+\delta_{ik}\right)-q/M\right),\Delta_{i,i}=\delta_{i,i}=0,\forall i\in K$. The inner sum of exponential terms can be written as

(A18) $$\begin{array}{c} \sum_{n=0}^{N-1}e^{-j\gamma_{i,l}^{p}n}=\left\{\begin{array}{c} \frac{1-e^{-j\gamma_{i,l}^{p}N}}{1-e^{-j\gamma_{i,l}^{p}}},p\neq N\Delta f\left(\tau_{i,l}+\Delta_{i,k}\right),\\ N,\;\;\;\;\;p=N\Delta f(\tau_{i,l}+\Delta_{i,k}). \end{array}\right.\\ \sum_{m=0}^{M-1}e^{j\gamma_{i,l}^{q}m}=\left\{\begin{array}{c} \frac{1-e^{j\gamma_{i,l}^{q}M}}{1-e^{j\gamma_{i,l}^{q}}},\;q\neq MT_{\mathrm{sym}}\left(\nu_{i,l}+\delta_{ik}\right),\\ M,\;\;\;\;\;q=MT_{\mathrm{sym}}\left(\nu_{i,l}+\delta_{ik}\right). \end{array}\right. \end{array}$$

Next, a digital LPF matrix G is applied to ${\tilde{F}}_{2}\left\{{\hat{H}}_{k}\right\}$ and is given by

(A19) $$G_{p,q+\frac{M}{2}}=\left\{\begin{array}{cc} 1, & \mathrm{if}\;|p|\leq P_{\mathrm{filt}}\;\mathrm{and}\;\left|q\right|\leq Q_{\mathrm{filt}},\\ 0, & \mathrm{otherwise}. \end{array}\right.$$

where ⌈x⌉ denotes the minimum integer no less than *x* and $P_{\mathrm{filt}}≜P_{\max}+P_{g},Q_{\mathrm{filt}}≜Q_{\max}+Q_{g},P_{\max}≜\left\lceil N\Delta f\tau_{k,\max}\right\rceil ,Q_{\max}≜\left\lceil MT_{\mathrm{sym}}\nu_{k,\max}\right\rceil$. $P_{g},Q_{g}>0$ denotes the designed guard interval of the filter in the two dimensions, respectively. For the analysis of the elimination of the interference, we focus on the power of the IUI-SEI component processed after the filter within the region $\left|p\right|\leq P_{\mathrm{filt}},\left|q\right|\leq Q_{\mathrm{filt}}$, which is given by

(A20) $$\begin{array}{cc} {\left|{\left[{\tilde{F}}_{2}\left\{{\tilde{H}}_{k}\right\}\cdot G\right]}_{p,q+\frac{M}{2}}\right|}^{2}\; & =\frac{1}{N^{2}M^{2}}\sum_{i=0,i\neq k}^{K}\sum_{l=0}^{L_{i}-1}{\left|\alpha_{i,l}\right|}^{2}\cdot {\left|\frac{1-e^{-j\gamma_{i,l}^{p}N}}{1-e^{-j\gamma_{i,l}^{p}}}\right|}^{2}{\left|\frac{1-e^{j\gamma_{i,l}^{q}M}}{1-e^{j\gamma_{i,l}^{q}}}\right|}^{2}. \end{array}$$

Without loss of generality, we focus on the analysis with k=0,i=1,…,K−1. We assume that $\tau_{k,\max}=\tau_{\max},\nu_{k,\max}=\nu_{\max},\forall k$. Additionally, the number of offsets between the user *i* and the user 0 is designed as follows based on the condition in Equation (11)

(A21) $$P_{i}^{\Delta }=iP_{\max}+{\tilde{P}}_{g}\;\mathrm{and}\;\;Q_{i}^{\delta }=0,\;i=1,\ldots ,K-1,$$

or

(A22) $$Q_{i}^{\delta }=2iQ_{\max}+{\tilde{Q}}_{g}\;\mathrm{and}\;\;P_{i}^{\Delta }=0,\;i=1,\ldots ,K-1,$$

where ${\tilde{P}}_{g}>P_{g},{\tilde{Q}}_{g}>Q_{g}$ is the designed guard interval of the offset in the two dimensions, respectively. For the following discussions, we first investigate the case with (A21).

To analyze the expression in (A20), we analyze the property of the function $f\left(\gamma \right)≜\left|\frac{1-e^{-j\gamma N}}{1-e^{-j\gamma }}\right|$ regarding $\gamma \in \left[0,...,\frac{2\pi p}{N},...,2\pi \right],p\in \left[0,1,...N\right]$. Firstly, since it is shown in (A23) that f(π+x)=f(π−x) for x∈[0,π], f(γ) is symmetric about π.

(A23) $$\begin{array}{c} f\left(\pi +x\right)-f\left(\pi -x\right)=\left|\frac{1-e^{-jxN}}{1+e^{-jx}}\right|-\left|\frac{1-e^{jxN}}{1+e^{jx}}\right|=0. \end{array}$$

Secondly, since sin(γN/2)=sin(πp) and $f\left(\gamma \right)=\left|\frac{1-e^{-j\gamma N}}{1-e^{-j\gamma }}\right|=\left|\frac{e^{-j\gamma N/2}\left(e^{j\gamma N/2}-e^{-j\gamma N/2}\right)}{e^{-j\gamma /2}\left(e^{j\gamma /2}-e^{-j\gamma /2}\right)}\right|=\left|\frac{\sin(\gamma N/2)}{\sin(\gamma /2)}\right|$, we can calculate that

(A24) $$\begin{array}{cc} f(\gamma (p+1))-f(\gamma (p)) & =\left|\frac{1}{\sin(\frac{\pi (p+1)}{N})}\right|-\left|\frac{1}{\sin(\frac{\pi p}{N})}\right|=\frac{\left|\sin(\frac{\pi p}{N})\right|-\left|\sin(\frac{\pi (p+1)}{N})\right|}{\left|\sin(\frac{\pi p}{N})\right|\left|\sin(\frac{\pi (p+1)}{N})\right|}. \end{array}$$

For the numerator $g\left(p\right)≜\left|\sin(\frac{\pi p}{N})\right|-\left|\sin(\frac{\pi (p+1)}{N})\right|$ in (A24), it is obvious that $g\left(p\right)<0,p\in \left(0,\frac{\pi }{2}\right)$ and that $g\left(p\right)>0,p\in \left(\frac{\pi }{2},\pi \right)$.

Then, due to the symmetry about π, we can conclude that f(γ) is monotonically decreasing on (0,π) and monotonically increasing on [π,2π]. The power of f(γ) with regard to the $\gamma_{i,l}^{p}$ in the delay domain under the simulation parameters in Table 1 in Section 4.1 is illustrated in Figure A1. It can be observed that the power decays by 40 dB with $\gamma^{p}\geq 0.05\pi$, and decays by more than 50 dB with $\gamma^{p}\geq 0.25\pi$. Therefore, with a proper design of *N* and Δf, it can be ensured that $|f\left(2\Delta f\tau_{\max}\right){|}^{2}$ is very small, indicating that the energy corresponding to a path of the user *i* in (A20) decays a lot from $p≜\lceil N\Delta f\tau_{i,l}\rceil$ to $p≜\left\lceil N\Delta f\tau_{i,l}\right\rceil \pm P_{\max}$ with a shift of $P_{\max}$ points in the discrete DD domain.

Next, we come back to the analysis of the power given in (A20) under the condition in (A21). It indicates that within the region $\left|p\right|\leq P_{\mathrm{filt}},\left|q\right|\leq Q_{\mathrm{filt}}$, we have that $\forall l\in \{0,1,..,L_{i}\}$

(A25) $$\begin{array}{cc} 2\pi (\Delta f\tau_{i,l}+\left(\left(i-1\right)P_{\max}+{\tilde{P}}_{g}-P_{g}\right)/N)\leq & \gamma_{i,l}^{p}\leq 2\pi \left(\Delta f\tau_{i,l}+\left(iP_{\max}+{\tilde{P}}_{g}\right)/N\right),\\ 2\pi (T_{\mathrm{sym}}\nu_{i,l}-Q_{\mathrm{filt}}/M)\leq & \gamma_{i,l}^{q}\leq 2\pi \left(T_{\mathrm{sym}}\nu_{i,l}+Q_{\mathrm{filt}}/M\right). \end{array}$$

Therefore, according to the properties of f(γ) analyzed above and the fact that $\alpha_{i,0}>\alpha_{i,1}>\ldots >\alpha_{i,L_{i}-1}$, we have that for $\left|p\right|\leq P_{\mathrm{filt}},\left|q\right|\leq Q_{\mathrm{filt}}$,

(A26) $$\begin{array}{cc} \; & P_{\mathrm{leak}}^{\max}≜\max\left\{{\left|{\left[{\tilde{F}}_{2}\left\{{\tilde{H}}_{k}\right\}\cdot G\right]}_{p,q+\frac{M}{2}}\right|}^{2}\right\}\\ \; & \;\;\;=\max\left\{{\left|{\left[{\tilde{F}}_{2}\left\{{\tilde{H}}_{k}\right\}\cdot G\right]}_{P_{\mathrm{filt}},Q_{1,0}+\frac{M}{2}}\right|}^{2},{\left|{\left[{\tilde{F}}_{2}\left\{{\tilde{H}}_{k}\right\}\cdot G\right]}_{0,Q_{K-1,L_{K-1}}+\frac{M}{2}}\right|}^{2}\right\}, \end{array}$$

where $Q_{i,l}≜\left\lceil MT_{\mathrm{sym}}\nu_{i,l}\right\rceil$. Therefore, if the guard interval of the offset and the filter in the delay dimension, i.e., ${\tilde{P}}_{g},P_{g}$, are designed properly, then the power of the IUI SEI component in the region of interest, i.e., $\left|p\right|\leq P_{\mathrm{filt}},\left|q\right|\leq Q_{\mathrm{filt}}$, for extracting the local SEI on the RD map can be ignored approximately under the condition in (A21). Likewise, since the properties of f(γ) still hold in the case with the Doppler domain, the same conclusion can be drawn and the proof is omitted here.

For example, for a 3-user multiple access, based on the simulation parameters given in Section 4.1 under a typical scenario, we can have that $P_{\max}\geq 200,Q_{\max}\geq 60$. To estimate the local SEI of user 0, the component of user 2 can be omitted since the power of the component corresponding to $|f\left(\gamma_{2,0}^{P_{\mathrm{filt}}}\right){|}^{2}$ decays by more than 40 dB. Therefore, we can design that ${\tilde{P}}_{g}=40,P_{g}=20,{\tilde{Q}}_{g}=20,Q_{g}=10$ to ensure that $P_{\mathrm{leak}}^{\max}$ is small enough to be omitted.

In conclusion, under the orthogonality condition in Equation (11), the IUI-SEI component can be effectively eliminated in the DD domain within the region of interest as defined by the LPF with the parameters properly designed.
